# Anesthetic management for surgery in a nemaline myopathy patient with difficult airway: A CARE-compliant case report

**DOI:** 10.1097/MD.0000000000036174

**Published:** 2023-11-17

**Authors:** Hoon Jung, Hyunjee Kim, See Woo Lee

**Affiliations:** a Department of Anesthesiology and Pain Medicine, School of Medicine, Kyungpook National University, Daegu, Republic of Korea.

**Keywords:** airway management, endotracheal intubation, general anesthesia, nemaline myopathy, neuromuscular blocking agents

## Abstract

**Rationale:**

Nemaline myopathy (NM) is a congenital disease characterized by nonprogressive or slowly progressing muscle weakness and may increase the risk of anesthesia in case of respiratory muscle or cardiac involvement. Care should be taken to prevent respiratory failure after surgery.

**Patient concerns:**

A 35-year-old man with NM, who had difficult airway, restrictive ventilatory pattern, and pulmonary hypertension, required general anesthesia for surgery because of limited mouth opening.

**Diagnoses:**

The patient was diagnosed with NM (*ACTA1* mutation) and coronoid hyperplasia.

**Interventions:**

Awake fiberoptic nasal intubation was performed following preparations for analgesia. General anesthesia was maintained using inhalational anesthetics and opioids without using neuromuscular blocking agents.

**Outcomes:**

General anesthesia remained well maintained during surgery, with no movement or spontaneous breathing of the patient and he recovered from anesthesia uneventfully without complications.

**Lessons:**

This report highlights the safe performance of anesthesia induction and recovery in a case where anesthesia management is necessary for surgery in a patient of NM at a high risk of anesthesia-related complications.

## 1. Introduction

General anesthesia requires neuromuscular blockade, and neuromuscular blocking agents are administered for this purpose. Inhalational anesthetics also have a neuromuscular blocking effect. For this reason, patients with myopathy require special attention if undergoing surgery under general anesthesia, to ensure that respiratory failure does not occur during postoperative recovery.

Nemaline myopathy (NM) is a congenital disease characterized by nonprogressive or slowly progressing muscle weakness and encompasses a group of genetically and clinically heterogeneous disabilities with varying severities. Clinical subtypes have been proposed based on patient spectra, comprising age of onset, types of genetic mutations, and severity of symptoms.^[1,2]^ In typical cases, muscle weakness primarily affects the neck flexors and proximal facial and limb muscles, with delayed distal involvement in some cases, and respiratory failure is the most common cause of death.^[1]^ Anesthetic considerations for surgery in patients with NM include a difficult airway (due to patients’ distinct facial features), poor respiratory reserve, and cardiac dysfunction. Additionally, preventing postoperative respiratory failure is a vital concern.

We encountered a clinical case of a patient with NM with grade 2/5 proximal muscle weakness, restrictive pulmonary pattern, and pulmonary hypertension, who required general anesthesia for coronoidectomy surgery because of limited mouth opening. Here, we describe our anesthesia approach, considering the patient’s vulnerability to anesthesia-related complications, and review the relevant literature.

## 2. Case report

A 35-year-old man (166 cm, 57 kg) diagnosed with NM was admitted to our hospital. The patient had no family history of NM but showed a developmental delay in early childhood. He began walking at age 3 years, had difficulty running from age 7 years, and had bilateral limb weakness from age 9 years. The patient could not hike and developed subjective dyspnea from the age of 16 years. From age 19 years, his speech was slurred. At age 20 years, the patient underwent echocardiography because of severe dyspnea, and was diagnosed with right ventricular outflow tract obstruction and cardiomegaly. Subsequently, bilateral limb weakness was exacerbated further to the point of difficulty in raising the shoulders; he could raise his legs but could not maintain this position. At age 28 years, the patient underwent a muscle biopsy and was diagnosed with NM (*ACTA1* mutation). Muscle weakness progressed further, and the patient began using a wheelchair at the age of 34 years owing to difficulty in ambulating. In addition, he developed intermittent dysphagia. He presented to the hospital with an exacerbating mouth-opening limitation and was diagnosed with bilateral coronoid hyperplasia. Following this, bilateral temporomandibular joint interpositional gap arthroplasty with a temporoparietal fascial flap and bilateral coronoidectomy under general anesthesia were scheduled owing to mouth opening limitations, as the patient showed a maximum mouth opening of 10 mm.

The preoperative assessment revealed anterolateral lead ST depression on an electrocardiogram. Two-dimensional echocardiography findings strongly suggested right ventricular hypertrophy and pulmonary hypertension: ejection fraction, 54%; moderate tricuspid regurgitation; right ventricular systolic pressure, 58.0 mm Hg; mild pulmonary regurgitation; dilated pulmonary trunk; dilated right atrial cavity, inferior vena cava dilatation and plethora; and pericardial effusion.^[3,4]^ Cardiac catheterization was not performed. Chest computed tomography confirmed muscle atrophy of the chest wall and cardiomegaly, and pulmonary function tests showed patterns of restrictive ventilatory defect. Muscle strength testing^[5]^ showed severe proximal limb weakness of grade 2 bilaterally (muscle moves only if the resistance of gravity is removed), grade 4 for distal upper limbs bilaterally (muscle strength is reduced but muscle contraction can still move joint against resistance), and grade 3 for distal lower limbs bilaterally (muscle strength is further reduced such that the joint can be moved only against gravity with the examiner’s resistance completely removed).

For surgery, nasotracheal intubation was required. Awake fiberoptic nasal intubation was planned because of his severely limited mouth opening. In the operating room, the patient’s vital signs were monitored, including blood pressure (BP), pulse oximetry, electrocardiogram, train of four (TOF), body temperature, and patient state index (PSI), and the BP and pulse readings were 131/86 mm Hg and 71 beats/min, respectively. Midazolam 1.5 mg and glycopyrrolate 0.1 mg were intravenously administered to reduce the patient’s anxiety before the surgery and reduce airway secretions. A bilateral superior laryngeal nerve block was performed at the level of the greater cornu of the hyoid bone by administering 2 mL of 2% lidocaine following a negative aspiration test. Transtracheal injection was performed with 2 ml of 4% lidocaine via the cricothyroid membrane. In addition, 2 puffs each of 10% lidocaine spray were applied to the nasal mucosa. A bronchoscope was inserted through the patient’s nasal cavity and passed through the vocal cord, and after confirming placement in the trachea, a nasotracheal tube was advanced gently to complete endotracheal intubation. During the intubation procedure, the patient did not demonstrate pain or gag reflex, but his pulse was elevated, for which esmolol 5 mg was intravenously administered. He was given intravenous propofol 60 mg immediately after completing intubation, and anesthesia was maintained with sevoflurane 1.5 to 2.2 vol% and intravenous remifentanil (target effect-site concentration of 1.5–2.0 μg/mL). Upon confirming the loss of spontaneous respiration, mechanical ventilation was maintained at a tidal volume of 440 to 470 mL, respiratory rate of 11 to 12 breaths/min, positive end-expiratory pressure (PEEP) of 5 mm Hg, airway pressure of 15 to 16 mm Hg, and a fraction of inspired oxygen of 0.5. An invasive arterial line was inserted in the right radial artery, and stroke volume variation was continuously calculated using the FloTrac-Vigileo® device (software V03.02, Edwards Lifesciences, Irvine, CA). Additionally, a central line was inserted through the right subclavian vein before beginning the surgery. The surgery lasted for 145 minutes without using a neuromuscular blocking agent, and TOF was maintained at 4/90. During surgery, the patient did not exhibit any movements or spontaneous respiration while on mechanical ventilation, and he showed stable PSI, BP, and pulse. Though intraoperative blood loss was not substantial, BP slowly declined; hence, phenylephrine 20 to 35 mcg/min was continuously infused, and the patient remained hemodynamically stable throughout the surgery.

Upon completion of surgery, sevoflurane and remifentanil infusions were stopped, and the patient resumed spontaneous breathing 5 minutes later. However, his tidal volume was below 150 mL and was irregular. Spontaneous respiration further improved 30 minutes after surgery, with a tidal volume of 500 to 1000 mL and a respiration rate of 8 to 10. Following this, the patient was extubated, with arterial blood gas analysis findings of pH 7.37, PCO_2_ 46 mm Hg, PO_2_ 117 mm Hg, and O_2_ saturation 99.2%. Subsequently, the patient was placed in his preferred left lateral decubitus position, and he remained in the operating room for approximately 20 minutes for further monitoring of his ventilation and hemodynamic status. He was then transferred to the intensive care unit, where the patient could maintain spontaneous breathing with a 2 L O_2_ supply via nasal cannula without pulmonary aspiration. On postoperative day 1, the arterial blood gas analysis findings were pH 45, PCO_2_ 38 mm Hg, PO_2_ 139 mm Hg, and O_2_ saturation 99.9%. As the patient showed no cardiopulmonary complications, he was transferred to the general ward and discharged on postoperative day 7.

## 3. Discussion

In addition to the adverse effects of using neuromuscular blocking agents for myopathies, NM also carries an increased risk of general anesthesia-related complications. A previous study reported that patients with NM rarely have cardiac involvement, and even those with cardiac involvement had only mild involvement.^[6]^ However, a more recent review stated that heart diseases are not uncommon among patients with NM, and several heart conditions have been noted, including hypertrophic cardiomyopathy, dilated cardiomyopathy, heart failure without cardiomyopathy, and arrhythmias; further, when respiratory muscles are involved, pulmonary hypertension and right ventricular heart failure may also occur secondarily.^[7]^ In our case, the patient showed right ventricular hypertrophy and pulmonary hypertension owing to cardiac involvement of NM. In addition, he exhibited overall weakness of the respiratory muscles and patterns of restrictive ventilatory defect. Although he could not walk because of muscle weakness, he displayed symptoms of dyspnea even in daily life while using a wheelchair. Such accompanying cardiopulmonary dysfunctions require careful hemodynamic management during the administration of general anesthesia.

In NM, characteristic facial features may develop as a result of head and neck muscle weakness; these features include an elongated face, high-arched palate and tented upper lip, displaced jaw, and small mouth, and complicate the process of tracheal intubation for general anesthesia. In addition to the above features, our patient developed severely limited mouth opening due to coronoid hyperplasia. An elongated face due to NM is considered to be one of the causes of mouth-opening limitation.^[8]^ Since the patient could only open his mouth by 10 mm, surgery required nasal intubation to secure a surgical field, and hence fiberoptic-guided nasal intubation had to be performed. Several considerations need to be made for smooth and safe fiberoptic awake nasotracheal intubation in patients with right ventricular hypertrophy, pulmonary hypertension, and restrictive lung patterns.

Pulmonary hypertension is defined as a mean pulmonary arterial pressure of ≥ 25 mm Hg as measured via right cardiac catheterization, and there has been a recent proposal to lower the criterion to ≥ 20 mm Hg.^[9]^ In our case, definitive cardiac catheterization was not performed because the patient refused; we, therefore, focused on preventing an exacerbation of pulmonary hypertension during anesthesia management based on echocardiography findings that strongly suggested pulmonary hypertension^[3,4]^ During anesthesia of patients with pulmonary hypertension, it is essential to maintain right ventricular cardiac output and avoid causing systemic hypotension.^[10,11]^ Maintaining right ventricular cardiac output requires maintaining an adequate preload, systemic vascular resistance, and contractility. In particular, it is crucial to avoid situations in which pulmonary hypertensive crisis attributed to increased right ventricular afterload, that is, factors that increase pulmonary vascular resistance, which include hypoxia, hypercarbia, acidosis, agitation, pain, and hypothermia. Excessive PEEP and high plateau pressure must be avoided during positive pressure ventilation, as they may contribute to pulmonary vascular resistance and right ventricular functions. Hypotension must be aggressively treated owing to the consequent reduction in right ventricular coronary perfusion, which is managed by systemic vasoconstrictors such as phenylephrine or vasopressin. In the present case, our patient was administered midazolam, transtracheal injection of local anesthetic, local anesthetic spray on the nasal mucosa, and nerve blockade to prevent increased pulmonary vascular resistance due to the patient’s anxiety and pain during fiberoptic awake nasal intubation. During the intubation procedure, as the patient’s pulse rate increased, esmolol was given to control it. Subsequently, the patient’s BP dropped during surgery, and phenylephrine, which increases systemic vascular resistance, was administered to maintain the BP while monitoring the patient’s volume status.

Restrictive lung patterns in our patient appeared to be the result of muscular atrophy and loss of muscle tone due to myopathy. Although there are no general recommendations for mechanical ventilation in restrictive pulmonary dysfunction, applying PEEP may be beneficial in preventing loss of functional residual capacity and improving ventilation by maintaining alveolar patency, and low tidal volume may also be helpful when lung compliance is poor.^[12]^ In this case, there was no parenchymal lung disease, and the primary management goal was to avoid an increase in pulmonary hypertension; thus, the tidal volume was set at approximately 7 mL/kg based on the ideal body weight^[13]^ and a lower PEEP of 5 mm Hg applied while care was taken not to trigger the development of hypercapnia and excessive airway pressure.

Among congenital myopathies, genetic linkage to a *RYR1* mutation, which is associated with malignant hyperthermia susceptibility, has been investigated.^[14]^ However, patients with NM have a very low risk for malignant hyperthermia, and our case corresponds to the *ACTA1* mutation, which is known to be unrelated to malignant hyperthermia susceptibility.^[14]^ Studies recommend avoiding the use of triggers for malignant hyperthermia, such as succinylcholine and volatile anesthetics, during general anesthesia in suspected NM patients who have not undergone genetic testing.^[15]^ Hence, total intravenous anesthesia is recommended in such cases. In our case, we used volatile inhalational anesthetics based on the genetic test results of an *ACTA1* mutation. Additionally, a previous report described successful use of volatile inhalational anesthetics in a patient with NM.^[16]^ Volatile inhalational anesthetics have neuromuscular blocking effects, making them clinically useful for anesthesia maintenance when neuromuscular blocking agents cannot be administered, as in our case.

Our patient had severe proximal muscle weakness with strength grade 2/5, and could not ambulate since a year before the presentation. In addition, he had chest wall muscle atrophy due to respiratory muscle involvement, showed a pattern of restrictive pulmonary dysfunction, and had dyspnea despite being in a wheelchair. To minimize the risk of postoperative respiratory failure, we withheld the use of neuromuscular blocking agents. Avoiding the use of neuromuscular blocking agents during endotracheal intubation may cause complications, such as airway injury or postoperative sore throat.^[17]^ However, the clinical pharmacokinetics of neuromuscular blocking agents may be altered in certain neuromuscular disorders such as myopathies and myasthenia gravis. Thus, avoiding neuromuscular blocking agents may be the reasonable choice in cases where determining the appropriate drug dose or achieving reversal of the drug effect is difficult. In our case, anesthesia was well maintained, without movement or spontaneous breathing of the patient during mechanical positive pressure ventilation, even though the TOF level was consistently high owing to the administration of only inhalational anesthetic and remifentanil. This is speculated to be attributable to the substantial loss of muscle tone as a result of severe muscle weakness caused by NM. Fortunately, the PSI value was maintained at an adequate level during surgery.

## 4. Conclusion

We described the case of a patient with NM with severe mouth-opening limitation and muscle weakness, pulmonary hypertension, and restrictive ventilatory defect patterns, who underwent awake fiberoptic nasotracheal intubation for surgery under general anesthesia that was induced and maintained without the use of neuromuscular blocking agents. As the *ACTA1* mutation observed in our patient is known not to be associated with malignant hyperthermia, we maintained anesthesia with sevoflurane and continuous infusion of remifentanil. The patient did not show any movement or spontaneous breathing during surgery even though no neuromuscular blocking agents were used. Additional precaution was taken to prevent any increase in pulmonary hypertension and manage mechanical ventilation. Using our approach, the patient remained hemodynamically stable during surgery and recovered from anesthesia uneventfully, without developing postoperative complications such as respiratory failure. This case illustrates the successful and safe administration of general anesthesia and uneventful recovery from anesthesia in a patient with NM at high risk of anesthetic complications owing to a difficult airway, severe muscle weakness, and cardiopulmonary insufficiency.

## Author contributions

**Conceptualization:** Hyunjee Kim, See Woo Lee.

**Supervision:** Hoon Jung.

**Writing – original draft:** Hyunjee Kim, See Woo Lee.

**Writing – review & editing:** Hyunjee Kim, See Woo Lee.
